# Genotyping with a 198 Mutation Arrayed Primer Extension Array for Hereditary Hearing Loss: Assessment of Its Diagnostic Value for Medical Practice

**DOI:** 10.1371/journal.pone.0011804

**Published:** 2010-07-26

**Authors:** Juan Rodriguez-Paris, Lynn Pique, Tahl Colen, Joseph Roberson, Phyllis Gardner, Iris Schrijver

**Affiliations:** 1 Department of Pathology, Stanford University School of Medicine, Stanford, California, United States of America; 2 California Ear Institute, Palo Alto, California, United States of America; 3 Department of Medicine, Stanford University School of Medicine, Stanford, California, United States of America; 4 Department of Pediatrics, Stanford University School of Medicine, Stanford, California, United States of America; Ohio State University Medical Center, United States of America

## Abstract

Molecular diagnostic testing of individuals with congenital sensorineural hearing loss typically begins with DNA sequencing of the *GJB2* gene. If the cause of the hearing loss is not identified in *GJB2*, additional testing can be ordered. However, the step-wise analysis of several genes often results in a protracted diagnostic process. The more comprehensive Hereditary Hearing Loss Arrayed Primer Extension microarray enables analysis of 198 mutations across eight genes (*GJB2*, *GJB6*, *GJB3*, *GJA1*, *SLC26A4*, *SLC26A5*, *MTRNR1* and *MTTS1*) in a single test. To evaluate the added diagnostic value of this microarray for our ethnically diverse patient population, we tested 144 individuals with congenital sensorineural hearing loss who were negative for biallelic *GJB2* or *GJB6* mutations. The array successfully detected all *GJB2* changes previously identified in the study group, confirming excellent assay performance. Additional mutations were identified in the *SLC26A4*, *SLC26A5* and *MTRNR1* genes of 12/144 individuals (8.3%), four of whom (2.8%) had genotypes consistent with pathogenicity. These results suggest that the current format of this microarray falls short of adding diagnostic value beyond the customary testing of *GJB2*, perhaps reflecting the array's limitations on the number of mutations included for each gene, but more likely resulting from unknown genetic contributors to this phenotype. We conclude that mutations in other hearing loss associated genes should be incorporated in the array as knowledge of the etiology of hearing loss evolves. Such future modification of the flexible configuration of the Hereditary Hearing Loss Arrayed Primer Extension microarray would improve its impact as a diagnostic tool.

## Introduction

Hearing loss is a common birth defect and the most prevalent sensory disorder affecting newborns in developed nations. The estimated incidence of permanent hearing loss at birth, defined in the U.S. as a sensorineural loss of 35 dB or more, is nearly one per 500 newborns [1]. The causes of hearing loss may be genetic, environmental or multifactorial. However, at least 50% of prelingual hearing loss in industrialized countries is of genetic origin [2]. Hearing loss can be non-syndromic when present in isolation (∼70%) or syndromic when accompanied by additional clinical features (∼30%) [3]. It can be inherited as a recessive, dominant, X-linked or mitochondrial trait, with autosomal recessive inheritance comprising approximately 80% of non-syndromic sensorineural hearing loss (NSNHL). Different mutations within the same gene can result in either dominant or recessive inheritance, further increasing the genetic complexity of this condition. As many as 300 human genes are thought to be involved in the process of hearing [4] and to date, more than 100 mapped or characterized loci have been implicated in NSNHL (Hereditary Hearing Loss home page: http://webh01.ua.ac.be/hhh/).

Current recommendations for molecular diagnostic testing of patients with congenital hearing loss advise to first test the *GJB2* gene that encodes the gap junction protein connexin 26. If the molecular basis of the hearing loss was not identified, this test may be followed by analyses of other genes, such as the del(*GJB6*-D13S1830) deletion in *GJB6* that encodes connexin 30 [2]. These two genes are adjacently located at the DFNB1 locus of chromosome 13q12, where mutations have been detected in as many as 50% of patients with autosomal recessive NSNHL [5]. However, a retrospective study in a large North American cohort reported that only 24.3% of patients diagnosed with NSNHL carried *GJB2* sequence changes [6]. Furthermore, biallelic *GJB2* variants were identified in just 11.2% of cases and the del(*GJB6*-D13S1830) deletion in *GJB6* was present in only 2.8% of individuals monoallelic for *GJB2* mutations. These results suggest that genes in loci other than DFNB1 may play a considerable role in the etiology of NSNHL.

In order to provide a faster, more comprehensive and cost-effective assay for NSNHL mutations, the HHL APEX (Hereditary Hearing Loss Arrayed Primer Extension) microarray was developed [7]. The APEX array comprises a panel of 198 sensorineural hearing loss mutations in six nuclear genes (*GJB2*, *GJB6*, *GJB3*, *GJA1*, *SLC26A4* and *SLC26A5*) and two mitochondrial genes (*MTRNR1* and *MTTS1*). APEX array technology facilitates the simultaneous analysis of multiple mutations through hybridization of fragmented template DNA to specifically designed primers, followed by single nucleotide extension at the site of each putative mutation [8], [9]. We wished to determine whether the HHL APEX microarray provides added diagnostic value beyond the standard testing of *GJB2*. Consequently, we studied 144 individuals with NSNHL in whom only one recessive *GJB2* mutation, one del(*GJB6*-D13S1830) deletion or no mutations had been identified by clinical genotyping. Our goal was to not only evaluate the APEX array as a diagnostic tool but also to further elucidate the contribution of the genes on the array to the development of congenital NSNHL.

## Materials and Methods

### Study subjects

This study was conducted at Stanford University with 144 participating individuals, mostly children, diagnosed with congenital NSNHL. All participants were probands, and none of their family members were enrolled in this study. Participants were either recruited at the California Ear Institute or at Stanford University under IRB approval with informed consent. Prior to enrollment, all participants had been tested for mutations in the *GJB2* gene by DNA sequencing as part of their routine clinical care. Some had received additional testing by PCR for the del(*GJB6*-D13S1830) deletion. To assess the added clinical diagnostic value of the HHL APEX microarray in its present configuration, this study included individuals for whom only one recessive *GJB2* mutation, one del(*GJB6*-D13S1830) deletion, or no mutations had previously been identified. Patients with a clear environmental cause for their hearing loss (such as significant noise exposure, a history of trauma, ototoxic medications, intrauterine infection, tumor or other known condition that affects hearing) were excluded. Although an environmental contribution cannot be entirely ruled out in any individual with hearing loss, it is unlikely to be a significant factor in any of our probands. Individuals with unequivocal syndromic hearing loss were also excluded from this study.

### Microarray Design

The APEX microarray (Asper Biotech) includes 198 mutations selected from six nuclear genes (*GJB2*, *GJB6*, *GJB3*, *GJA1*, *SLC26A4* and *SLC26A5*) and two mitochondrial genes (*MTRNR1* and *MTTS1*); a detailed list of sequence variants is provided in Table S1. These genes were selected because they have been implicated in sensorineural hearing loss [7]. The mutation list encompasses single nucleotide changes as well as insertions and deletions, including the large del(*GJB6*-D13S1830) deletion which truncates the *GJB6* gene. For each of the 198 mutations on the array, 25 bp oligonucleotides were constructed in both the sense and antisense directions, based on the wild-type consensus sequence for each gene (www.ncbi.nlm.nih.gov/Genbank/). These were spotted in duplicate onto each microarray slide. Most oligonucleotides were designed to be extended by 1 bp, directly at the site of the mutation, allowing accurate detection of the nucleotide change. However, for those deletions and insertions for which this extension resulted in the same nucleotide as was present in the wild-type DNA, optimal discrimination was achieved by oligonucleotides extending further into the deletion or insertion.

### Genomic template preparation

Genomic DNA was extracted from saliva using the Oragene® DNA self-collection kit (DNA Genotek, Inc) according to the manufacturer's protocol, or from peripheral blood following standard procedures. Genomic regions of interest were first PCR amplified and purified and then enzymatically treated with uracil *N*-glycosylase and shrimp alkaline phosphatase to concurrently fragment the DNA and inactivate unincorporated dinucleotide triphosphates.

### APEX reactions and analysis

APEX was performed as previously described [7]. Briefly, fragmented genomic template DNA was denatured, mixed with Thermo Sequenase DNA Polymerase (USB Corporation) and fluorescently labeled dideoxynucleotides before being applied to microarray slides for a 20 minute isothermic APEX reaction. After washing the slides, duplicate fluorescent signals for both complementary DNA strands were detected and recorded using a Genorama Quattroimager (Asper Biotech), thus producing four analytical data points corresponding to each mutation position. This approach reduces the likelihood of false positive signals while ensuring a clear distinction between homozygous and heterozygous sequence changes.

## Results

**Figure 1:**
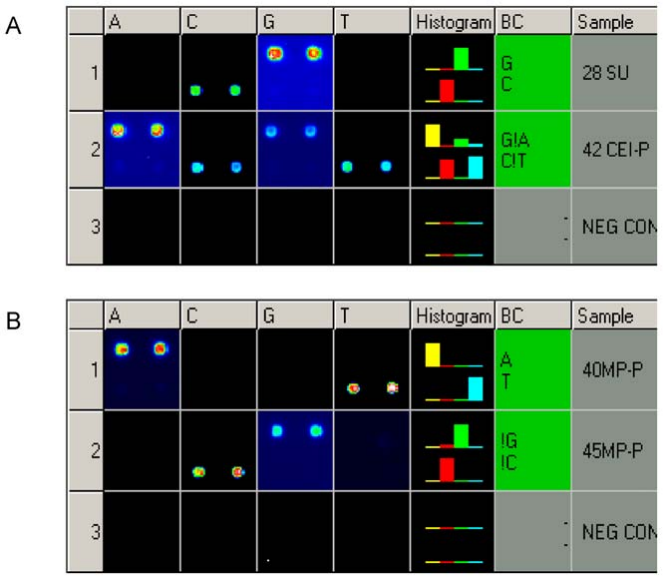
APEX detection of the W24X (71G>A) mutation in *GJB2* and the 1555A>G mutation in *MTRNR1*. A) W24X in GJB2. Row 1. Wild-type genotype for nucleotide position 71 in the *GJB2* gene. In the sense direction (S) the wild-type G allele is detected, and in the antisense (AS) direction the complementary C allele is identified. Row 2. Heterozygous for W24X. S: the wild-type G allele and the mutant A allele are both present. AS: the wild-type C allele and the mutant T allele are detected. Row 3. Negative control. B) 1555A>G in *MTRNR1*. Row 1. Wild-type genotype for nucleotide position 1555 in the MTRNR1 gene. The wild-type A allele is identified in S, and the complementary T allele is detected in AS. Row 2. Homoplasmic for 1555A>G. S: the mutant G allele is present. AS: the complementary mutant C allele is detected. Row 3. Negative control.

We sought to assess whether the HHL APEX array provides additional value as a diagnostic tool and to determine the frequencies of mutations included on the array in our participants with congenital NSNHL. If the subject's mutations are included on the APEX array, detection of two mutations would be expected when the hearing loss displays a recessive pattern of inheritance; these two mutations may either be homozygous and located at a single mutation site, or compound heterozygous with mutations at two different positions within a gene. If the patient has dominant sensorineural hearing loss, then a single dominant mutation would be expected. Representative APEX array results for two study subjects with congenital NSNHL are presented in Fig 1. A heterozygous W24X (71G>A) mutation was identified in the *GJB2* gene of a single patient (42 CEI-P, Fig 1A). Fig 1B depicts the homoplasmic mutation 1555A>G detected in the mitochondrial *MTRNR1* gene of a second patient (45 MP-P). Both mutations were identified in the forward and reverse sequence directions. No appreciable differences in APEX signal quality were observed when comparing assay results for templates amplified from genomic DNA derived from saliva versus results for templates derived from blood (data not shown).

The sequence variants detected by the microarray, including those few changes classified as benign by the Connexin-Deafness Homepage (http://davinci.crg.es/deafness/), are summarized in Table S1. Sequence changes were identified in 35.4% (51/144) of subjects; 8.3% (12/144) carried previously undetected mutations in the *SLC26A4*, *SLC26A5* and/or *MTRNR1* genes (Table 1). Mutations and changes of, as yet, unknown clinical significance in the pendrin gene (*SLC26A4*) were identified in nine subjects and encompassed six heterozygous, one homozygous and two compound heterozygous sequence changes. Of the six individuals in whom heterozygous *SLC26A4* mutations were discovered, three carried heterozygous sequence changes in other genes. One patient carried the benign R127H variant in *GJB2* as well as the L597S variant in *SLC26A4*; two others carried splice variant IVS2-2A>G (of unknown clinical significance) in the prestin gene (*SLC26A5*) with either the F335L or the V609G mutation in *SLC26A4*.

**Table 1:** Genotypes detected by APEX in 144 congenitally hearing impaired individuals with one or no previously identified *GJB2* or *GJB6* mutations.

Homozygous	(n)	Compound Heterozygous	(n)	Heterozygous	(n)	Multiple Sequence Variants	(n)
GJB2; V27I*/V27I*	5	GJB2; M34T#/333-334delAA	1	GJB2; G12V	1	GJB2; V27I*/V27I/E114G	2
(GJB2; V27I*)/SLC26A4; 919-2A>G/919-2A>G	1	SLC26A4; IVS1-2A>G**/L236P	1	GJB2; 35delG	2	GJB2; V27I*/V37I	1
		SLC26A4; N324Y/V609G	1	GJB2; W24X	1	GJB2; V27I*/E114G*	1
				GJB2; V27I*	13	(GJB2; V37I)/MTRNR1; 1555A>G†	1
				GJB2; M34T	2	GJB2; R127H*/SLC26A4; L597S**	1
				GJB2; V37I	3	SLC26A4; F335L/SLC26A5; IVS2-2A>G**	1
				GJB2; R127H*	2	SLC26A4; V609G/SLC26A5; IVS2-2A>G**	1
				GJB2; S139N	1		
				GJB2; R165W	1		
				GJB2; W172X	1		
				GJB2; I203T*	2		
				SLC26A4; G209V	1		
				SLC26A4; L597S**	1		
				SLC26A4; V609G	1		
				SLC26A5; IVS2-2A>G**	2		
							
	**6**		**3**	**Total**	**34**		**8**

*Benign Variant.

**Unknown clinical significance.

†Homoplasmic.

#Pathogenic with reduced penetrance; of unclear clinical significance at the time of study, which is why this patient was included. [30].

In addition to identifying nine individuals with sequence changes in the *SLC26A4* gene, the APEX array also detected sequence variants in the *SLC26A5* and *MTRNR1* genes of three others. Two carried the IVS2-2A>G splice variant in the *SLC26A5* gene. A third individual was homoplasmic for the 1555A>G mutation of the *MTRNR1* gene that encodes the 12S-rRNA in mitochondria (Fig 1). This individual also carried the V37I mutation in the *GJB2* gene. In total, the APEX array identified potentially pathogenic genotypes in only four patients (2.8%, 4/144): three were either homozygous or compound heterozygous for sequence changes in the nuclear *SLC26A4* gene and one was homoplasmic for the 1555A>G substitution in the mitochondrial *MTRNR1* gene. Changes in *GJB3*, *GJA1*, and *MTTS1* were not identified in any subjects.

## Discussion

The implementation of universal newborn hearing screening across the U.S. has resulted in early identification of hearing loss with benefits to language, social, and cognitive development [10]. The majority of affected children (∼70%) will have no other clinical manifestations, which precludes a diagnosis of the underlying etiology by clinical examination. Molecular diagnostic testing, typically starting with DNA sequence analysis of the *GJB2* gene, is frequently conducted to identify the underlying cause of the hearing loss, enabling accurate genetic counseling and guiding management and treatment. However, depending on the population studied, mutations in *GJB2* only provide a definitive explanation in up to half of the individuals with NSNHL. When a molecular cause is not identified by testing the *GJB2* gene, analysis of additional genes is frequently pursued, based on clinical and family history as well as cost considerations. Such testing is usually performed for one gene at a time, and the diagnostic process, even if successful, can span years. The HHL APEX array was developed to provide added value to the diagnostic process by testing eight different genes in a single test, thus drastically shortening the time to molecular diagnosis. The array includes all mutations reported for each included gene at the time of development of the assay. However, novel mutations in several genes, including *GJB2* and *SLC26A4*, continue to be discovered and are spread throughout the genes without true hot-spots. Lack of identification of mutations on the HHL array, therefore, does not necessarily exclude a gene as the cause of the hearing loss, illustrating an intrinsic disadvantage of any assay that does not examine the entire gene sequence. Ideally, clinical diagnostic testing with an APEX-based array would cover all known variants for the included genes and would not require follow-up sequencing in the majority of cases. However, until we have a more complete understanding of the mutation spectrum in individual genes, targeted diagnostic sequencing would be a follow-up tool to screening with the APEX assay, for individuals in whom a single pathogenic mutation was found in a gene on the array.

Our study group was comprised of individuals in whom only one recessive *GJB2* mutation, one del(*GJB6*-D13S1830) deletion or no mutations had previously been identified by clinical diagnostic testing. Given that *GJB2* and *GJB6* mutations are included as part of the APEX panel, a subset of the subjects was known to carry mutant alleles that should be confirmed by APEX testing. Of the 51 patient genotypes detected by the array, 42 included sequence changes in *GJB2*. The array results for these 42 individuals were consistent with the previous molecular diagnostic findings (data not shown), thus confirming the reported 100% sensitivity and reliability of the HHL APEX assay [7]. Mutations in the connexin gene family of gap junction proteins represent approximately half of the array and encompass sequence changes in the *GJB2* (connexin 26), *GJB6* (connexin 30), *GJB3* (connexin 31) and *GJA1* (connexin 43) genes. Mutations in *GJB2* are the most common known cause of autosomal recessive NSNHL in many populations [11], while the frequency of deletions in *GJB6* appears much more population-dependent [6]. Mutations in *GJB3* and *GJA1* were also chosen for inclusion on the microarray, based on early studies linking changes in these genes with NSNHL [12], [13]. Sequence changes in *GJB3* have been identified in individuals with hearing loss from several populations [14], but they are rare and their pathogenicity remains controversial. In addition, the mutations identified in *GJA1* that were associated with sensorineural hearing loss may actually be located in a pseudogene of the connexin 43-encoding *GJA1* gene, on chromosome 5 [15]. Considering that the APEX array did not detect a single *GJB3* or *GJA1* variant in any of our study subjects, and without further evidence implicating the involvement of these two genes in NSNHL, inclusion of *GJB3* or *GJA1* in future versions of the APEX array may not be warranted.

In addition to connexin mutations, the array includes sequence variants in the *SLC26A4* and *SLC26A5* genes, two members of the SLC26 family of anion transporters. *SLC26A5* has been implicated in non-syndromic SNHL [16] whereas *SLC26A4* gene mutations are associated with both autosomal recessive non-syndromic SNHL (DFNB4) and Pendred syndrome [17], [18]. Pendred syndrome is a relatively common form of syndromic deafness that often appears non-syndromic during childhood. The actual incidence is difficult to ascertain due to the late onset and incomplete penetrance of the characteristic euthyroid goiter [19]. Furthermore, without performing computed tomography or magnetic resonance imaging an enlarged vestibular aqueduct (EVA) or Mondini dysplasia, which are associated with Pendred syndrome but not pathognomonic, are not observable. Considering the phenotypic variability among patients with *SLC26A4* mutations and the difficulty of diagnosing Pendred syndrome, the frequency of sensorineural hearing loss resulting from DFNB4/Pendred syndrome may be underestimated [20]. At least one *SLC26A4* variant was detected by the APEX array in nine subjects. Unfortunately, temporal bone imaging results were not available on these patients. The array identified one *SLC26A4* homozygous (919-2A>G/919-2A>G) and two compound heterozygous genotypes (N324Y/V609G and IVS1-2A>G/L236P). Of note, whereas L236P is a frequently occurring *SLC26A4* mutation, accounting for 16% of Pendred disease-causing alleles [21], IVS1-2A>G is a rare splice variant, the clinical significance of which has yet to be established. In silico predictions, as well as discordant segregation with EVA in an affected fraternal twin pair suggest that this sequence change may not actually affect gene function or expression [22], [23]. Six individuals carried a single mutant *SLC26A4* allele, either alone or in combination with a mutant allele at a second gene locus. Although some studies do correlate hearing loss with non-syndromic EVA in patients carrying a single *SLC26A4* mutation [22], [23], the assumed mode of inheritance is autosomal recessive. The most plausible explanation for not detecting a second *SLC26A4* mutation in these individuals is that the mutation was not included on the APEX panel. This is not necessarily due to the method, however: even with full *SLC26A4* gene sequencing, only a single mutation is identified in a third of multiplex families with Pendred syndrome (http://www.ncbi.nlm.nih.gov/sites/GeneTests/?db=GeneTests). Four individuals were heterozygous for the IVS2-2A>G variant in *SLC26A5*. Similar to IVS1-2A>G in *SLC26A4*, its clinical significance is unknown. Expected to result in the skipping of exon 3 during RNA processing, it has been found at the same frequency in individuals with hearing loss and in a control group and pathogenicity seems now unlikely [24], [25]. Therefore, this gene would likely be excluded from future versions of the APEX assay. Finally, a single, homoplasmic mitochondrial mutation (1555A>G in the *MTRNR1* gene) was identified. Both mitochondrial genes on the array represent mutation hot-spot regions associated with NSHL [26]. The 1555A>G mutation predisposes affected individuals to irreversible hearing loss upon treatment with aminoglycosides.

Recently, two high-throughput resequencing arrays for mutations in SNHL-associated genes were developed by research groups from Harvard and from Cincinnati [27]. These Affymetrix-based arrays encompass a total of 13 genes that were selected based on published mutation frequencies, recessive inheritance patterns and severity of genetic impact on patient outcome. The HHL APEX array and the Affymetrix resequencing arrays both incorporate the *GJB2*, *GJB6*, *SLC26A4* and *SLC26A5* genes. Overall, however, the resequencing arrays represent a broader spectrum of SNHL genes, with more genes associated with syndromic hearing loss. In particular, three genes implicated in Type 1 Usher syndrome (*CDH23*, *MYO7A* and *USH1C*), while selected for the resequencing arrays, were not incorporated in the HHL APEX array; a separate APEX array for this group of disorders was already available when the HHL APEX array was originally designed [28]. In addition to compositional differences, the HHL APEX array and the Affymetrix resequencing arrays employ different assay methodologies. The HHL APEX array focuses specifically on the detection of previously reported hearing loss mutations whereas the Affymetrix resequencing arrays allow for the discovery of new mutations. However, resequencing arrays have reduced ability to detect small deletions and insertions, which constitute ∼24% of disease-causing mutations in the Human Gene Mutation Database [27]. The HHL APEX array was specifically designed to reliably detect such deletions and insertions. In addition, the Harvard and Cincinnati groups both reported a high false-positive rate (∼70%) among identified variants, thus requiring confirmatory sequencing which is time-consuming and adds cost [27].

Mutations in the *MYO15A*, *OTOF*, *TMC1* and other genes have emerged as being among the more prevalent nonsyndromic hearing loss mutations, worldwide [3]. These mutations were not included on the APEX microarray because, at the time of its design, only limited data were available regarding the role of these genes in hearing loss. Furthermore, the information available in the literature was restricted to small groups encompassing specific geographic populations [29]. As future versions of the APEX microarray are developed, consideration will be given to incorporating these genes in the APEX platform.

Although APEX array technology provides a sensitive and reliable platform for mutation detection, our investigation of the HHL APEX microarray as a relatively comprehensive assay for our multi-ethnic patient population revealed a lack of additional diagnostic value beyond standard molecular diagnostic testing of *GJB2*. Of 144 probands negative for biallelic *GJB2* and/or *GJB6* mutations, the HHL array identified previously unidentified sequence changes in only 12 individuals (8.3%) among whom four potentially pathogenic genotypes were found (2.8%). These results suggest the existence of previously unreported hearing loss mutations in the eight genes comprising the HHL APEX panel, and, more importantly, mutations in pathogenic loci not currently included. Since the APEX panel must necessarily be limited to a finite number of genes and sequence variants, the present array configuration may also not accurately represent the most frequent hearing loss alleles in a given test population. Further research will be required to characterize allele frequencies in distinct populations, and to identify new mutations in the hundreds of genes comprising the molecular hearing apparatus. The APEX microarray does offer a flexible platform that can be easily modified to alter mutation composition and/or to increase the number of mutations. Thus, it can be adjusted to our evolving understanding of the etiology of hearing loss and may be more clinically useful in a future configuration.

## Supporting Information

Table S1 Complete list of gDNA sequence variants detectable with the SNHL Apex array.
(0.30 MB DOC)
