# Supplementary material for: Genotyping with a 198 Mutation Arrayed Primer Extension Array for Hereditary Hearing Loss: Assessment of Its Diagnostic Value for Medical Practice
Source: PLoS One. 2010 Jul 26;5(7):e11804. doi: 10.1371/journal.pone.0011804 (PMC2909915; doi:10.1371/journal.pone.0011804)
Supplement: Table S1 — Complete list of gDNA sequence variants detectable with the SNHL Apex array. (0.30 MB DOC) [file pone.0011804.s001.doc]

**Table S1. Complete List of gDNA Sequence Variants Detectable with the SNHL Apex array**

| **Pathogenic** | | **Unknown Significance** | | **Benign** | |
| --- | --- | --- | --- | --- | --- |
| **Amino Acid Change** | **Nucleotide Change** | **Amino Acid Change** | **Nucleotide Change** | **Amino Acid Change** | **Nucleotide Change** |
| *GJB2* (connexin 26) nonsyndromic, recessive | | | | | |
| N/A | IVS1+1G>A | V27I+E114G | 79G>A + 341A>G | V27I | 79G>A |
| N/A | -3170G>A |  |  | E114G | 341A>G |
| M1V | 1A>G |  |  | R127H | 380G>A |
| T8M | 23C>T |  |  | I203T | 608T>C |
| Frameshift | 31del14 |  |  |  |  |
| Frameshift | 31del38 |  |  |  |  |
| G12V | 35G>T |  |  |  |  |
| Frameshift | 35delG |  |  |  |  |
| Frameshift | 35insG |  |  |  |  |
| K15T | 44A>C |  |  |  |  |
| Frameshift | 51del12insA |  |  |  |  |
| S19T | 56G>C |  |  |  |  |
| I20T | 59T>C |  |  |  |  |
| W24X | 71G>A |  |  |  |  |
| R32C | 94C>T |  |  |  |  |
| R32H | 95G>A |  |  |  |  |
| R32L | 95G>T |  |  |  |  |
| M34T | 101T>C |  |  |  |  |
| V37I | 109G>A |  |  |  |  |
| A40E | 119C>A |  |  |  |  |
| A40G | 119C>G |  |  |  |  |
| W44X | 132G>A |  |  |  |  |
| G45E | 134G>A |  |  |  |  |
| E47X | 139G>T |  |  |  |  |
| E47K | 139G>A |  |  |  |  |
| Frameshift | 167delT |  |  |  |  |
| Q57X | 169C>T |  |  |  |  |
| Frameshift | 176-191del16 |  |  |  |  |
| C64X | 192C>A |  |  |  |  |
| Y65X | 195C>G |  |  |  |  |
| W77R | 229T>C |  |  |  |  |
| W77X | 231G>A |  |  |  |  |
| Frameshift | 235delC |  |  |  |  |
| L79P | 236T>C |  |  |  |  |
| Q80X | 238C>T |  |  |  |  |
| Q80P | 239A>C |  |  |  |  |
| Q80R | 239A>G |  |  |  |  |
| I82M | 246C>G |  |  |  |  |
| V84L | 250G>C |  |  |  |  |
| S85P | 253T>C |  |  |  |  |
| A88S | 262G>T |  |  |  |  |
| L90V | 268C>G |  |  |  |  |
| L90P | 269T>C |  |  |  |  |
| Frameshift | 269insT |  |  |  |  |
| M93I | 279G>A |  |  |  |  |
| V95M | 283G>A |  |  |  |  |
| Y97X | 291C>A or C>G |  |  |  |  |
| Frameshift | 290-291insA |  |  |  |  |
| H100Y | 298C>T |  |  |  |  |
| Frameshift | 299-300delAT |  |  |  |  |
| H100L | 299A>T |  |  |  |  |
| DelK102 | 302delAGA |  |  |  |  |
| E101G | 302A>G |  |  |  |  |
| Frameshift | 310del14 |  |  |  |  |
| Frameshift | 312del14 |  |  |  |  |
| Frameshift | 314del14 |  |  |  |  |
| Frameshift | 333-334delAA |  |  |  |  |
| S113R | 339T>G |  |  |  |  |
| DelE120 | 360delGAG |  |  |  |  |
| K122I | 365A>T |  |  |  |  |
| Q124X | 370C>T |  |  |  |  |
| W133X | 398G>A |  |  |  |  |
| Y136X | 408C>A |  |  |  |  |
| S139N | 416G>A |  |  |  |  |
| R143W | 427C>T |  |  |  |  |
| E147K | 439G>A |  |  |  |  |
| E147X | 439G>T |  |  |  |  |
| Frameshift | 486insT |  |  |  |  |
| R165W | 493C>T |  |  |  |  |
| Frameshift | 504insAAGG |  |  |  |  |
| Frameshift | 509del14 |  |  |  |  |
| Frameshift | 509insA |  |  |  |  |
| Frameshift | 515del17 |  |  |  |  |
| W172X | 516G>A |  |  |  |  |
| C174R | 520T>C |  |  |  |  |
| P175T | 523C>T |  |  |  |  |
| V178A | 533T>C |  |  |  |  |
| R184W | 550C>G |  |  |  |  |
| R184P | 551G>C |  |  |  |  |
| Frameshift | 572delT |  |  |  |  |
| S199F | 596C>T |  |  |  |  |
| Frameshift | 605ins46 |  |  |  |  |
| I203K | 608TC>AA |  |  |  |  |
| N206S | 617A>G |  |  |  |  |
| Frameshift | 631delGT |  |  |  |  |
| L214P | 641T>C |  |  |  |  |
| Frameshift | 645-648delTAGA |  |  |  |  |
| *GJB2* (connexin 26) nonsyndromic, dominant | | | | | |
| DelE42 | 125delAGG |  |  |  |  |
| W44S | 131G>C |  |  |  |  |
| W44C | 132G>C |  |  |  |  |
| R75Q | 224G>A |  |  |  |  |
| M163L | 487A>C |  |  |  |  |
| D179N | 535G>A |  |  |  |  |
| R184Q | 551G>A |  |  |  |  |
| C202F | 605G>T |  |  |  |  |
| *GJB2* (connexin 26) syndromic, dominant | | | | | |
| G12R | 34G>C |  |  |  |  |
| S17F | 50C>T |  |  |  |  |
| D50N | 148G>A |  |  |  |  |
| N54K | 162C>A or C>G |  |  |  |  |
| G59A | 176G>C |  |  |  |  |
| D66H | 196G>C |  |  |  |  |
| R75W | 223C>T |  |  |  |  |
| R75Q | 224G>A |  |  |  |  |
| *GJB3* (connexin 31) nonsyndromic, recessive | | | | | |
|  |  | Del Ile141 | 421delATT |  |  |
|  |  | I141V | 421A>G |  |  |
|  |  | P223T | 667C>A |  |  |
| *GJB3* (connexin 31) nonsyndromic, dominant | | | | | |
| R180X | 538C>T |  |  |  |  |
| E183K | 547G>A |  |  |  |  |
| *GJB6* (connexin 30) nonsyndromic, recessive | | | | | |
| N/A | ~309 kb del |  |  |  |  |
| *GJB6* (connexin 30) nonsyndromic, dominant | | | | | |
| T5M | 14C>T |  |  |  |  |
| Frameshift | 63delG |  |  |  |  |
| *GJA1* (connexin 43) nonsyndromic, recessive | | | | | |
|  |  | L11F | 31C>T |  |  |
|  |  | V24A | 71T>C |  |  |
| Mitochondrial nonsyndromic | | | | | |
| *MTRNR1* | 1555A>G |  |  |  |  |
| *MTTS* | 7445A>G,C |  |  |  |  |
| *MTTS* | 7472insC |  |  |  |  |
| *MTTS* | 7511T>C |  |  |  |  |
| *SLC26A5* (Prestin) nonsyndromic, recessive | | | | | |
|  |  | N/A | IVS2-2A>G |  |  |
| *SLC26A4* (Prendrin) Pendred syndromic, recessive | | | | | |
| M1T | 2T>C | N/A | IVS1-2A>G |  |  |
| R24G | 70C>G | L597S | 1790T>C |  |  |
| S28R | 84C>A |  |  |  |  |
| N/A | IVS2-1G>A |  |  |  |  |
| Y78C | 233A>G |  |  |  |  |
| X96 | 279delT |  |  |  |  |
| A104V | 311C>T |  |  |  |  |
| Y105C | 314A>G |  |  |  |  |
| A106D | 317C>A |  |  |  |  |
| L117F | 349C>T |  |  |  |  |
| Frameshift | 336_377insT |  |  |  |  |
| T132I | 395C>T |  |  |  |  |
| S133T | 397T>A |  |  |  |  |
| Frameshift | 407_411delTCTCA |  |  |  |  |
| V138F | 412G>T |  |  |  |  |
| V138X | 25bp del + 5 bp ins |  |  |  |  |
| Frameshift | IVS4+7A>G |  |  |  |  |
| G139A | 416G>C |  |  |  |  |
| T193I | 578C>T |  |  |  |  |
| G209V | 626G>T |  |  |  |  |
| L236P | 707T>C |  |  |  |  |
| Frameshift | 753_756delCTCT |  |  |  |  |
| Frameshift | 783_784insT |  |  |  |  |
| D271H | 811G>C |  |  |  |  |
| Frameshift | 917delT |  |  |  |  |
| N/A | IVS7-2A>G |  |  |  |  |
| N/A | IVS7+1G>A |  |  |  |  |
| N/A | IVS8+1G>A |  |  |  |  |
| N/A | IVS8-2A>G |  |  |  |  |
| N324Y | 970A>T |  |  |  |  |
| F335L | 1003T>C |  |  |  |  |
| K369E | 1105A>G |  |  |  |  |
| A372V | 1115C>T |  |  |  |  |
| Frameshift | 1147delC |  |  |  |  |
| E384G | 1151A>G |  |  |  |  |
| S394del | 1181_3delTCT |  |  |  |  |
| Frameshift | 1197delT |  |  |  |  |
| R409H | 1226G>A |  |  |  |  |
| T410M | 1229C>T |  |  |  |  |
| A411P | 1231G>C |  |  |  |  |
| T416P | 1246A>C |  |  |  |  |
| Q421R | 1262A>G |  |  |  |  |
| N/A | 1264-1G>C |  |  |  |  |
| A429del | 1284_1286delTGC |  |  |  |  |
| L445W | 1334T>G |  |  |  |  |
| Frameshift | 1334_1335insAGTC |  |  |  |  |
| Q446R | 1337A>G |  |  |  |  |
| Frameshift | 1341delG |  |  |  |  |
| V480D | 1439T>A |  |  |  |  |
| I490L | 1468A>C |  |  |  |  |
| G497S | 1489G>A |  |  |  |  |
| T508N | 1523C>A |  |  |  |  |
| Frameshift | 1536_1537delAG |  |  |  |  |
| N/A | IVS13+9C>G |  |  |  |  |
| Y530H | 1588T>C |  |  |  |  |
| N/A | IVS14+1G>A |  |  |  |  |
| Y556H | 1666T>C |  |  |  |  |
| Y556C | 1667A>G |  |  |  |  |
| C565Y | 1694G>A |  |  |  |  |
| V609G | 1826T>G |  |  |  |  |
| Frameshift | 1898delA |  |  |  |  |
| V653A | 1958T>C |  |  |  |  |
| F667C | 2000T>G |  |  |  |  |
| G672E | 2015G>A |  |  |  |  |
| F683S | 2048T>C |  |  |  |  |
| Frameshift | 2111_2112insGCTGG |  |  |  |  |
| Frameshift | 2127delT |  |  |  |  |
| T721M | 2162C>T |  |  |  |  |
| H723R | 2168A>G |  |  |  |  |
| D724G | 2171A>G |  |  |  |  |
| Frameshift | 2182_2183insG |  |  |  |  |
| G740S | 2218G>A |  |  |  |  |
| X781W | 2343A>G |  |  |  |  |
